# Multi-objective green vehicle scheduling problem considering time window and emission factors in ship block transportation

**DOI:** 10.1038/s41598-024-61578-2

**Published:** 2024-05-11

**Authors:** Hui Guo, Jucheng Wang, Jing Sun, Xuezhang Mao

**Affiliations:** 1 School of Naval Architecture & Ocean Engineering, Jiangsu University of Science and Technology, Room 311, Science and Technology Building, Zhenjiang, Jiangsu Province People’s Republic of China; 2 Jiangsu Modern Shipbuilding Technology Co., Ltd, Zhenjiang, 212100 China

**Keywords:** Ship block transportation, Green shipbuilding, Green vehicle scheduling, Improved genetic whale optimization algorithm, Fuel consumption, Computational science, Computer science, Information technology

## Abstract

Logistics distribution is one of the main sources of carbon dioxide emissions at present, and there are also such distribution problems in the shipbuilding process. With the increasing attention paid to environmental problems, how to effectively reduce the energy consumption of block transportation and improve the utilization rate of resources in the factory is the key problem that China’s shipbuilding industry needs to solve at present. This article considers the time windows for block transportation tasks, as well as the self-loading constraints of different types of flat cars, and establishes an optimization model that minimizes the empty transport time and energy consumption of the flat cars as the optimization objective. Then, an Improved Genetic Whale Optimization Algorithm is designed, which combines the cross and mutation ideas of genetic algorithms and proposes a whale individual position updating mechanism under a mixed strategy. Furthermore, the performance and computational efficiency of the algorithm are verified through comparative analysis with other classical optimization algorithms on standard test examples. Finally, the shipyard’s block transportation example proves that the energy-saving ship block transportation scheduling method can effectively improve the efficiency of shipbuilding enterprise’s block transportation and reduce the energy consumption in the block transportation process. It proves the engineering practicality of the green dispatching method proposed in this paper, which can further provide a decision-making method for shipyard managers.

## Introduction

Logistics distribution activities are the most important among logistics activities, and also one of the activities with large energy consumption. The unreasonable transportation phenomena such as high empty driving rate, repeated transportation, and staggered transportation in distribution activities have aggravated air pollution and resource waste, and research shows that logistics distribution has become one of the main sources of carbon dioxide emissions^1^. Such distribution problems also exist in the shipbuilding process. With the continuous deepening of the China’s Double-Carbon policy^2^, promoting the greening of the shipbuilding process, effectively reducing the energy consumption in the manufacturing process, and improving the resource utilization rate are the key issues that need to be solved in China’s shipbuilding industry at present, and also have certain reference significance to help the shipbuilding industry achieve the “Double Carbon” goal.

Generally, the shipyard will have more than one ship built at the same time. Each ship is divided into hundreds of sections of different sizes. These sections not only need to be produced in the workshop but also need to be transported between the workshops and storage yards of the shipyard by flat cars. In the process of transportation in sections, unreasonable scheduling schemes will generate more energy consumption for transportation. Therefore, it is necessary to schedule the block transportation tasks reasonably, reduce the empty time of the flat car in the transportation process, reduce the energy consumption of the flat car, improve the block transportation efficiency, and reduce the cost of the enterprise.

However, some shipyards still use the relatively backward experience-based method in the block transportation scheduling, which leads to the block transportation efficiency is still not very high, and often there is a high no-load rate of vehicles, repetitive tasks, and other situations. This phenomenon is more prominent when building new ships, which will aggravate environmental pollution and cause the waste of enterprise resources. At present, domestic and foreign research on the scheduling problem of ship staging has achieved certain results^3–5^, but most of the existing scheduling models and methods focus on improving efficiency and saving time, which is difficult to deal with the multi-objective optimization scheduling problem of ship staging under the low-carbon target, which requires establishing new models and proposing new methods.

Logistics transportation is the main factor of carbon emissions. Due to the particularity of segmented transportation in shipyards, there are significant differences from general vehicle scheduling problems. Unreasonable scheduling schemes will generate more carbon emissions. However, most existing research only considers delivery time constraints, which cannot meet the current requirements of green and sustainable development. Therefore, based on previous research, this article establishes a mathematical model that is more in line with the actual needs of shipyards by considering carbon emissions and time windows. In the whole shipbuilding process, block transportation is a very important step, and it will also consume more resources. It is the key link to improving the efficiency of ship construction and reducing production energy consumption. Therefore, it is necessary to solve the block transportation scheduling problem for the low-carbon goal, to provide fast and accurate decision support for the management personnel related to ship construction and production, avoid the waste of resources in the process of ship block transportation, and ensure the stability and continuity of the ship block transportation process.

The remainder of the paper is organized as follows: Sect. “Literature review” reviews the related literature. Section “Problem description and formulation” presents a description of the problem and proposes a mathematical model. Section “Improved genetic whale optimization algorithm” gives the details of the improved genetic whale optimization algorithm. Computational results on numerous instances are reported in Sect. “Experimental Verification”. Conclusions and future research directions are suggested in Sect. “Conclusions”.

## Literature review

During the whole process of ship construction, each block needs to be rotated many times in different workshops or yards. Because the weight and volume of the blocks are large, the requirements for transportation are strict to prevent damage to the blocks, which leads to the low efficiency of the shipyard’s block transportation, which usually requires overtime scheduling, Therefore, how to schedule the block transportation tasks more reasonably and scientifically has attracted the attention of relevant researchers at home and abroad. For the flatcar transportation scheduling problem of hull block, it can be seen as a vehicle routing problem (VRP). However, unlike traditional VRP^6,7^, the starting and ending points of block transportation are different, which leads to more complex block transportation problems than general VRP problems, which are also NP-hard problems. As the tardiness will bring serious economic losses to shipyards, it can be regarded as a green vehicle routing problem with hard time windows (GVRP-TW).

With the application of modern shipbuilding models, some scholars have also begun to conduct research on optimizing ship blocks transportation scheduling. Lee^8^ studies the problem of identifying the working state of flat cars in the shipyard when they are moving irregularly around the site when they are not present. Tao et al.^9^ established a mathematical model with the optimization objective of minimizing the idle time, waiting time, and delay time of the flatbed truck. Li et al.^10^ studies the scheduling problem of multi-flatcar coordinated transportation. In order to actively respond to the call for green shipbuilding, Jiang et al.^11^ proposed the “Multiple Vehicles, One Cargo” (MVOC) green transportation scheduling problem.

Based on the comprehensive analysis of the above research on the ship block transportation scheduling problem, it can be found that for the existing research results, the corresponding problem model and solution method have been formed, but the existing model and method rarely consider the energy consumption problem generated by flatcars in the scheduling process. To solve the multi-objective scheduling problem of energy-saving oriented ship block transportation, it is necessary to study the corresponding solution models and methods.

With the increasing attention paid to environmental protection, researchers pay more and more attention to fuel consumption and carbon emissions during vehicle transportation^12–14^. In recent years, more and more scholars^14–16^ take the carbon emissions in the process of vehicle transportation as one of the optimization objectives, establish the corresponding scheduling model and solve it. Nayera et al.^17^ proposed Random Green VRP (GVRP) that considers three objectives: economic, environmental, and social, and proposed and validated a new hybrid search algorithm to solve the VRP problem. Zeynep et al.^18^ proposed an interactive fuzzy method to solve the green capacity vehicle routing problem with imprecise travel time requirements for each vehicle and supplier. Cheng et al.^19^ developed a mixed integer programming model to minimize the total carbon footprint and penalty cost of delayed orders in efficient fishbone warehouse layouts with dynamic arrival orders and transportation time constraints in joint order batch processing and picking routing problems. Behnamian et al.^20^ proposed a mathematical model for the green heterogeneous vehicle routing problem and developed the firefly algorithm in large-scale examples to solve the problem. Chen et al.^21^ studied the cold chain green multi warehouse vehicle routing problem (CC-GMD-VRPTW-MF) with time windows and mixed fleets for urban logistics distribution using electric vehicles (EVs) and gasoline diesel vehicles (GDVs). Xiao et al.^22^ established a multi-objective comprehensive model of green simultaneous delivery under fuzzy demand to minimize the total cost composed of service cost, fuel consumption cost, and carbon emission cost. For the multi-site vehicle path problem under time-varying road networks, Fan et al.^23^ proposed an integer planning model with the minimum total cost and designed a hybrid genetic algorithm with variable neighbourhood search for this problem. Other scholars^24–26^ have considered the carbon emissions during vehicle driving when studying the vehicle scheduling problem. They have established corresponding scheduling models and designed different intelligent optimization algorithms to solve the problem model.

The above article studied the vehicle routing problem considering energy conservation. However, due to the significant differences between ship block transportation problems and general vehicle scheduling problems, firstly, ship block transportation has a larger volume and weight compared to other goods, which requires higher transportation requirements. Secondly, ship block processing has strict plans, and delays will incur significant costs. It is necessary to strictly adhere to time windows. The most important point is that the starting and ending points of ship block transportation are not the same, which is the biggest characteristic of ship block transportation problems. Therefore, the above results cannot be directly applied to segmented transportation scheduling. Therefore, it is necessary to establish a flatbed truck transportation scheduling problem model that considers energy consumption and design corresponding algorithms.

## Problem description and formulation

### Energy consumption analysis of ship section transportation process

As shown in Table 1, according to the relevant data of a shipyard, the fuel consumption of different types of flatbed cars in the month is quite different, which not only indicates that the shipyard has not reasonably scheduled different types of flatbed cars, but also indicates that this unreasonable scheduling scheme will generate large energy consumption, increase the economic cost of the enterprise, and increase the environmental burden.

**Table 1:** Monthly fuel consumption of platform trailers in a shipyard.

Vehicle type	Number	Oil consumption (L)
100t flat car	1#	543
200t flat car	2#	2001
200t flat car	3#	1424
100t flat car	4#	1535
150t flat car	5#	2566
600t flat car	6#	0
150t flat car	7#	1934

For the green vehicle scheduling problem, there are many different calculation models. In this paper, the more widely used Load Based Fuel Consumption Model (LFCM)^27^ is adopted, that is, the energy consumption of vehicle transportation has a certain correlation with the total vehicle weight. The fuel consumption per unit distance of a flat car is defined as the fuel consumption rate (FCR). The relationship is as follows:

1 $$ X = 0.0000793W - 0.026 $$

where W represents the weight of the flat car, kg; X represents fuel consumption rate, km/kg.

Without losing generality, when the flat car transports goods, W in the above formula can be expressed as two parts of vehicle weight $Q_{0}$ and segment weight $Q_{1}$, and the fuel consumption rate $\rho \left( {Q_{1} } \right)$ can be expressed as a linear function, namely:

2 $$ \rho \left( {Q_{1} } \right) = a\left( {Q_{0} + Q_{1} } \right) + b $$

where *a* and *b* are calculation parameters.

Suppose *Q* is the maximum load capacity of the flat car, and use $\rho^{*}$ to express the fuel consumption rate of the flat car when it is fully loaded; In the same way, $\rho_{0}$ is used to indicate the fuel consumption rate when the flat car is empty; According to formula (2):

3 $$ \left\{ {\begin{array}{*{20}l} {\rho_{0} = \alpha Q_{0} + b} \hfill \\ {\rho^{*} = \alpha \left( {Q_{0} + Q} \right) + b} \hfill \\ \end{array} \Rightarrow \alpha = \frac{{\rho^{*} - \rho_{0} }}{Q}} \right. $$

Therefore, $\rho \left( {Q_{1} } \right)$ can be written as:

4 $$ \rho \left( {Q_{1} } \right) = \rho_{0} + \frac{{\rho^{*} - \rho_{0} }}{Q}Q_{1} $$

### Problem description

**Figure 1:**
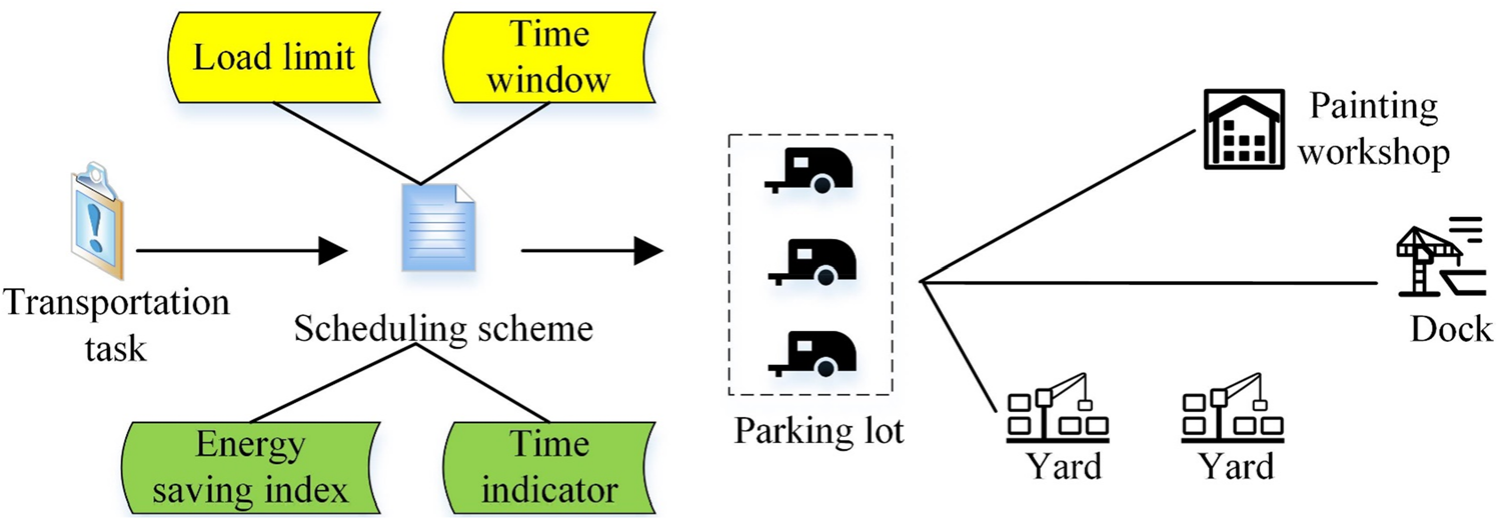
Green scheduling process of ship block transportation.

Taking the actual situation of a shipyard as an example, the ship block transportation scheduling problem considering the energy consumption of flat cars is described as follows: suppose that a shipyard currently has n sections to be transported to different locations, and there are m flat cars with different loads that the shipyard can carry out transportation tasks. For each block transportation task, a flat car meeting the load limit needs to be selected from the flat car set to carry out the transportation task. Due to the limited space of the block workshop, the block manufacturing workshop must be transported out as soon as possible after processing a segment, otherwise, it will affect the storage of the next segment after processing. At the same time, it is also necessary to transport the section to the subsequent operation site on time to ensure that the next operation process is carried out on time by the section, that is, the delivery time needs to meet the requirements of the time window, to ensure the continuity of section production and section transportation, and avoid conflicts between the two stages. The dispatching process of ship section transportation is shown in Fig. 1.

**Figure 2:**
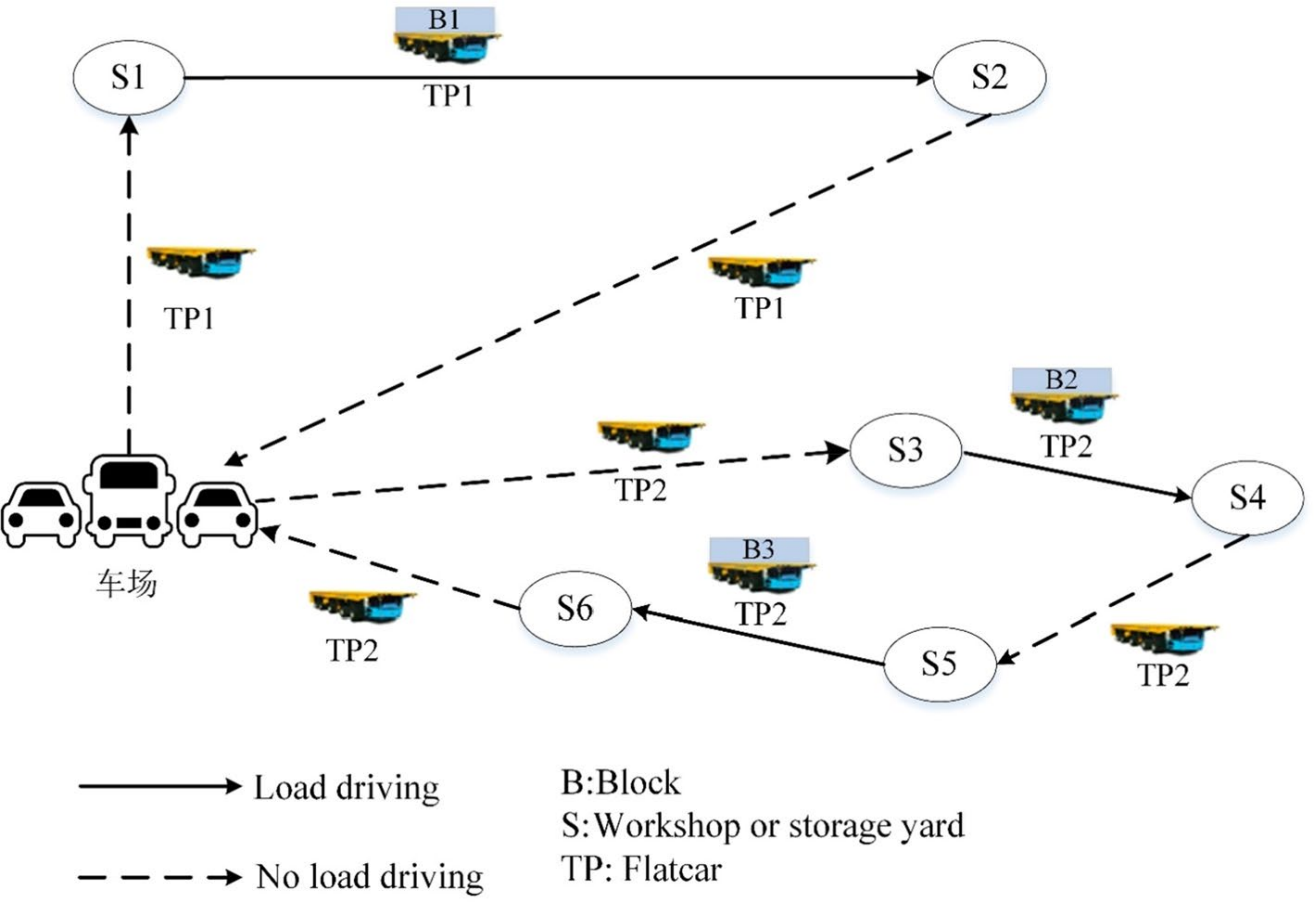
Problem description.

As shown in Fig. 2, it is assumed that there are currently two flatcars that need to complete three block transportation tasks. According to the plan, flatcar TP1 starts from the parking lot and travels with no load to the starting point S1 of task 1, transports block B1 with load to the destination S2 and then returns to the parking lot with no load. On the other side, the flatcar TP2 drives from the parking lot to the starting point S3 of task 2 with no load, drives block B2 loaded to destination S4, then drives to the starting point S5 of task 3 with no load, drives block B3 loaded to destination S6 and finally returns to the parking lot with no load.

### Assumptions

Before the further study of the problem, to facilitate the research of the problem and the establishment and solution of the model, the following assumptions are made:^11^.

1. Due to the load limitation of the flatcar, each flatcar can only load one block per task;
2. Once the flatcar starts to perform the transportation task, it will not be disturbed;
3. The speed of the flatcar when performing tasks is certain;
4. When the flatcar performs the task, it thinks that the road is not blocked;
5. It is assumed that the flatcar will not fail during transportation.

### Mathematical model

Suppose a shipyard has *n* block transportation tasks every day, and each block transportation task contains the following attributes as shown in Table 2:

**Table 2:** Parameter setting.

Variable	Meaning
i	Task no., $i = 1,2,3, \cdots ,n$
m	Flatcar no., $m = 1,2,3, \cdots ,M$
$Sp_{0}$	Parking lot
$d\left( {Sp_{i} ,Ep_{i} } \right)$	The transportation distance of task* i* from the starting point to the destination
$\left( {St_{i} ,Et_{i} } \right)$	The time window allowed for task* i*
$Rt_{i}$	Actual start time of task *i*
$Ut_{i}$	Service time of task *i,* block loading and unloading time at the task location
$Nt_{ij}$	It refers to the no-load time when the flat car drives to the task location* j* after completing task *i*, when $i = 0$, it means the flatcar starts from the parking lot, and when $j = 0$, it means the flatcar starts from the parking lot or returns to the parking lot, where $i \ne j$;
$Lt_{i}$	Indicates the load travel time of the flat car when it travels from the starting point of task *i* to the destination of task* i*
$w_{i}$	Weight of task *i*, unit: ton
$W_{j}$	The load capacity of flatcar* j*
R	A large integer
$\rho_{0}$	Fuel consumption per unit distance when flatcar is unloaded
$\rho^{*}$	Fuel consumption per unit distance when flatcar is fully loaded
$\rho$	Fuel consumption per unit distance when flatcar transports task *i*
$\omega_{1}$	Weight of flatcar’s no-load travel time
$\omega_{2}$	Weight of flatcar energy consumption

The perfect situation for block transportation scheduling is that the flatcar arrives at the location of task* i* before the time window allowed by task *i*, and then loads in the block. It transports block *i* to the destination before the deadline of the time window, finishes unloading, and then drives to the destination with no load in front of the time window allowed by the next task.

Set the following variables to describe the prepared block transportation scheduling scheme. For the *i*th block transportation task, there are:

5 $$ x_{im} = \left\{ {\begin{array}{*{20}l} {1{ }} \hfill & {{\text{Task }}i{\text{ is transported by flatcar }}m} \hfill \\ 0 \hfill & {{\text{other}}} \hfill \\ \end{array} } \right. $$

6 $$ y_{imj} = \left\{ {\begin{array}{*{20}l} 1 \hfill & {{\text{Flatcar }}m{\text{ transports task }}j{\text{ after transportation task }}i} \hfill \\ 0 \hfill & {{\text{other}}} \hfill \\ \end{array} } \right. $$

Specifically, when $i = 0$, $y\_0mj$ indicates that task *j* is the first transportation task of flatcar *m*; When $j = 0$, $y\_im0$ indicates that task* i* is the last task of flatcar *m*.

For a block transportation task, the starting point and ending point are determined, so the time of block transportation is determined. However, since the position of the flatcar when it goes to the starting point of the next task is uncertain, the no-load time of the flatcar changes. Similarly, the energy consumption of the flatcar transportation process will change with different scheduling schemes. Therefore, the sum of the empty time of flatcars that have completed all block transportation tasks and the energy consumption of flatcars are taken as the optimization objective, namely:

1. No load time of flat car.
  The no-load time of the flatcar is the time when the flatcar moves from its current position to the starting point of the next block transportation task when it executes the next block transportation task and the no-load transportation time when the flatcar completes the task and returns to the depot. In particular, when the first transport task of the flatcar is task *i*, its no-load time is the time when the flatcar travels from the parking lot to the starting point of the task.
  7 $$ f_{1} = \mathop \sum \limits_{m = 1}^{M} \mathop \sum \limits_{j = 1,j \ne i}^{n} \mathop \sum \limits_{i = 1}^{n} y_{imj} \cdot Nt_{ij} + \mathop \sum \limits_{m = 1}^{M} \mathop \sum \limits_{j = 1}^{n} y_{0mj} \cdot Nt_{0j} + \mathop \sum \limits_{m = 1}^{M} \mathop \sum \limits_{i = 1}^{n} y_{im0} \cdot Nt_{i0} $$
2. Vehicle energy consumption, including energy consumption during no-load operation and normal transportation.
  8 $$ \rho = \rho_{0} + \frac{{\rho^{*} - \rho_{0} }}{{W_{j} }}w_{i} $$
  9 $$ f_{3} = \mathop \sum \limits_{i \in n} min\left( {\rho_{0} d\left( {Sp_{0} ,Ep_{i} } \right) + \rho d\left( {Sp_{i} ,Ep_{i} } \right)} \right) $$

To facilitate optimization and reduce the amount of calculation, it is also convenient for dispatchers to generate corresponding scheduling schemes according to different situations, and give different weights to the two objective functions to combine them into a single objective function:

10 $$ F = \omega_{1} * f_{1} + \omega_{2} * f_{2} $$

11 $$ \omega_{1} + \omega_{2} = 1 $$

In formula (10), $\omega_{1}$, $\omega_{2}$ represents the weight coefficients of the two objective functions respectively. Since the two objective functions represent different indicators respectively, they may be in different orders of magnitude during calculation. If a certain objective value is too large, the final optimization result will be affected. Therefore, the normalization method proposed by Katragjini et al.^28^is adopted for processing, so that the final result range can be guaranteed to fall within the [0, 1] range. The normalized objective function expression is as follows:

12 $$ F = \omega_{1} * N\left( {f_{1} } \right) + \omega_{2} * N\left( {f_{2} } \right) $$

Including:

13 $$ N\left( {f_{1} } \right) = \frac{{F - \left( {f_{1} } \right)_{min} }}{{\left( {f_{1} } \right)_{max} - \left( {f_{1} } \right)_{min} }} $$

14 $$ N\left( {f_{2} } \right) = \frac{{F - \left( {f_{2} } \right)_{min} }}{{\left( {f_{2} } \right)_{max} - \left( {f_{2} } \right)_{min} }} $$

In Formula (13) and (14), $\left( {f_{i} } \right)_{max}$ and $\left( {f_{i} } \right)_{min}$ represents the upper and lower bounds of the objective function respectively.

The constraints are:

1. For two adjacent tasks of the same flatcar, time constraints need to be met as follows:
  15 $$ Rt_{i} - Rt_{j} + Ut_{i} + LT_{i} + y_{imj} \cdot Nt_{ij} \le R \cdot \left( {1 - \mathop \sum \limits_{m \in M} y_{imj} } \right)\quad \forall i,m,j $$
2. The weight of the block shall not exceed the load limit of the flatcar.
  16 $$ \mathop \sum \limits_{i \in N} x_{im} \cdot w_{i} \le W_{m} $$
3. Each flatcar has one and only one first task.
  17 $$ y_{0mj} = 1 $$
4. The actual start time of task *i* shall be within the specified time window.
  18 $$ St_{i} \le Rt_{i} \le Et_{i} \quad 3\forall i \in n $$
5. A task can only be executed once by the assigned flatcar.

19 $$ \mathop \sum \limits_{{\begin{array}{*{20}c} {i \in n} \\ {i \ne j} \\ \end{array} }} y_{imj} + y_{0mj} = x_{jm} \quad \forall j,m $$

20 $$ \mathop \sum \limits_{{\begin{array}{*{20}c} {j \in n} \\ {j \ne i} \\ \end{array} }} y_{imj} + y_{imo} = y_{il} ,\quad \forall i,m $$

21 $$ \mathop \sum \limits_{{\begin{array}{*{20}c} {j \in n} \\ {j \ne i} \\ \end{array} }} y_{imj} + y_{imo} = y_{il} ,\quad \forall i,m $$

## Improved genetic whale optimization algorithm

The whale optimization algorithm (WOA)^29^ is a new meta-heuristic algorithm based on swarm intelligence in recent years. The algorithm has the advantages of strong global search ability, fast convergence, few control parameters, easy implementation, etc. It has an excellent performance in solving optimization problems and has a wide range of applications. At present, it has been effectively applied in feature selection^30^, machine learning^31^, clustering^32,33^, power dispatching^34,35^, etc.

Since the ship block transportation scheduling problem considering the energy consumption of flat cars is a typical NP problem, it is difficult to directly obtain an accurate solution. According to relevant research, the whale optimization algorithm has a good performance in solving the vehicle scheduling problem^36^. Therefore, this paper proposes an Improved Genetic Whale Optimization Algorithm (IGWOA). IGWOA is described in detail below.

### Chromosome coding and decoding

The ship block transportation scheduling problem mainly includes two aspects: transportation task allocation and scheduling order. Therefore, the method of double coding is adopted, and chromosomes are set as positive integers to form a two-dimensional array, representing the transportation task order and flatcar scheduling order respectively. The first line represents the sequence of block transportation tasks, the number in the *i-th* position represents the serial number of transportation tasks, the second line represents the scheduling sequence of flatcars, and the number in the *i-th* position represents the number of flatcars that perform the corresponding tasks. Through this relationship, the transportation tasks and flatcar numbers corresponded one by one.

**Figure 3:**
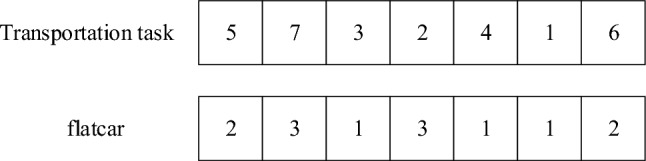
Coding method.

Suppose 3 flatcars are performing 7 transportation tasks, and the chromosomes are shown in Fig. 3. The decoding process is to map the flatcar sequence to the transportation task one by one. The chromosomes in the figure can be represented by decoding as follows: 1# flatcar needs to perform transportation task 3, task 4, and task 1, 2# flatcar needs to perform transportation task 5 and task 6, and 3# flatcar needs to perform transportation task 7 and task 2.

### Chaos map population initialization

First, the transportation task sequence is generated by using the random method, and then the flatcar sequence is generated by considering the load capacity constraints, that is, randomly selecting one flatcar from the flatcars that meet the load requirements of the block transportation task.

For the swarm intelligent optimization algorithm, the initial population with good quality can effectively speed up the algorithm’s solving speed and obtain the optimal solution with high quality. Therefore, the diversity of the initial population should be improved as much as possible when initializing the population. However, the standard WOA algorithm uses the method of producing random numbers within a certain range to generate the initial population. This method leads to poor diversity of the initial population, which is easy to concentrate in a certain range, and it is easy to fall into the local optimum during the search iteration, with low efficiency. On the contrary, Chaotic Maps have good randomness and ergodicity and can search the solution space more comprehensively. Therefore, the characteristics of chaotic mapping are used to make up for the shortcomings of the basic whale algorithm and generate the initial population. Gaganpreet et al.^37^ used different Chaotic Maps to optimize WOA and finally concluded that the tent map is the best among all mapping methods studied, which can effectively improve the performance of the WOA algorithm. Therefore, when initializing the population, we use Tent mapping.

First, mapping the initial whale position variable to the definition domain [0, 1] of Tent mapping, and the formula is as follows:

22 $$ z_{i} = \frac{{x_{0} - l_{b} }}{{u_{b} - l_{b} }} $$

Then, using Tent mapping to generate chaotic variables, the formula is as follows:

23 $$ z_{i + 1} = \left\{ {\begin{array}{*{20}l} {\frac{{z_{i} }}{0.7}} \hfill & {z_{i} < 0.7}\\ \hfill \\ {\frac{10}{3}\left( {1 - z_{i} } \right)} \hfill & {z_{i} \ge 0.7} \hfill \\ \end{array} } \right. $$

Finally, the chaotic variable is transformed into a whale position variable through inverse mapping, and the formula is as follows:

24 $$ x_{i} = l_{b} + \left( {u_{b} - l_{b} } \right)z_{i} $$

where $l_{b}$ and $u_{b}$ are the minimum and maximum values of the optimization variable interval, $x_{i}$ is the whale position variable, and $z_{i}$ is the chaotic variable.

### Crossover and mutation operation

To reduce the repair operations after general mutation, this chapter selects the Subtour Exchange Crossover (SEX) mode.

**Figure 4:**
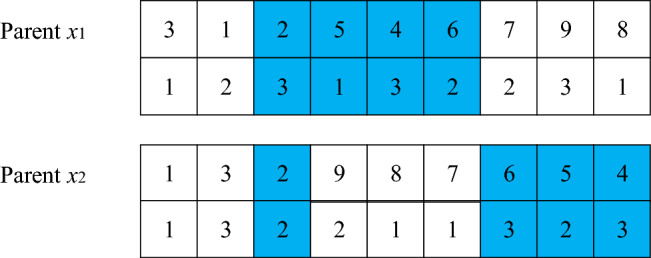
First step of crossing.

**Figure 5:**
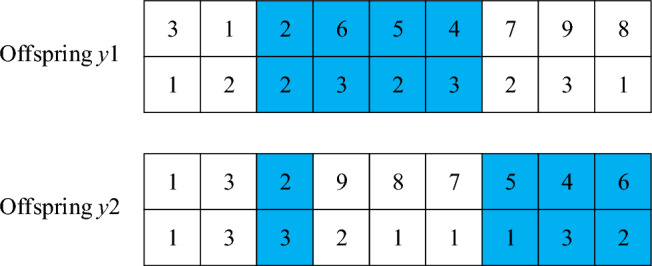
Second step of crossing.
  Step 1: first calculate the length of chromosomes, and then generate two random integers within the length range, such as 3 and 6. Select gene fragments (2, 5, 4, 6) at positions 3–6 on the parent generation ${ }x_{1}$, and find the positions of these genes in the parent generation $x_{2}$.
  Step 2: fix the position of the unselected genes, and then exchange the genes in the parent chromosome according to the order of the selected genes in the chromosome, so that two child chromosomes can be directly generated.
- Mutation operation
  During chromosome mutation operation, select the method of cross mutation, that is, randomly select two points, such as 3 and 6. If only the task sequence is mutated, the mutated task may exceed the maximum load capacity of the flatcar. Therefore, to ensure the constraint conditions between the task and the flatcar, the flat car serial numbers corresponding to the task are exchanged and mutated together to obtain sub-chromosomes, as shown in Fig. 6.

- SEX crossover
  The fitness function is calculated according to the initial population, and the chromosome with the largest fitness value is selected as the global optimal solution by comparison. The crossover process is shown in Figs. 4 and 5, which can be described as follows: randomly select a chromosome from the current population as the crossover parent $x_{1} = \left( {3,1,2,5,4,6,7,9,8} \right)$, and then select the current global optimal solution as the parent $x_{2} = \left( {1,3,2,9,8,7,6,5,4} \right)$. $x_{1}$ and $x_{2}$ cannot be the same.

**Figure 6:**
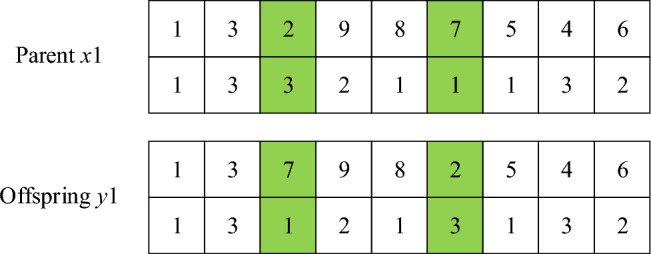
Variation operation.

### Updating mechanism of whale individual position under hybrid strategy

The basic WOA adjusts and balances the exploration and development capability of the algorithm through parameter *A*. However, since the change of parameter a is linear, it cannot effectively adjust the convergence rate of the algorithm. As for the WOA algorithm, it is required to have better global exploration ability in the early stage of iteration to avoid falling into the local optimum early. In the middle stage of the algorithm iteration, with the gradual deepening of global exploration, it is necessary to accelerate the convergence speed. In the late stage of the algorithm iteration, with the gradual convergence of global exploration, the scope of the optimal solution is determined. It is necessary to slow down the speed, carry out the local search, and improve the quality of the optimal solution and the accuracy of the algorithm. Therefore, considering the needs of different stages, an updated formula for the convergence factor *a* of the stage is proposed, as shown below:

25 $$ \left\{ {\begin{array}{*{20}l} {a = 2 - e^{{ - \frac{i}{Max\_iter}}} } \hfill & {i \le \frac{1}{2}Max\_iter} \hfill \\ {a = 1 - e^{{\left( {\frac{i}{Max\_iter} - 1} \right)}} } \hfill & {i > \frac{1}{2}Max\_iter} \hfill \\ \end{array} } \right. $$

To avoid premature convergence of the algorithm, the adaptive weight factor is introduced into the position update formula. Most studies usually use linear adjustment of inertia weight. Although it is simple and intuitive, it cannot fully coordinate the global and local search performance of the algorithm. To increase the searchability of whales, this chapter proposes a dynamic inertia weight factor $w$ and introduces it to the whale position update method. The specific formula is as follows:

26 $$ \vec{X}\left( {t + 1} \right) = w\vec{X}\left( t \right) - \vec{A} \cdot \vec{D},p < 0.5,\left| A \right| \le 1 $$

27 $$ \vec{X}\left( {t + 1} \right) = w\vec{D} \cdot e^{\omega t} \cdot \cos \left( {2\pi l} \right) + \overrightarrow {{X^{*} }} \left( t \right),p \ge 0.5 $$

28 $$ \vec{X}\left( {t + 1} \right) = w\vec{X}_{{\text{rand }}} \left( t \right) - \vec{A} \cdot \vec{D}_{{\text{rand }}} p\left\langle {0.5,\left| A \right|} \right\rangle 1 $$

29 $$ w = e^{{\frac{{ - 4.5^{*} i}}{{Max_{ - } iter}}}} $$

where *i* is the number of current iterations of the algorithm, and *Max_iter* is the maximum number of iterations set for the algorithm.

The adaptive weight factor can better search the search space because it maintains a large value in the early stage of the algorithm. In the later stage of the algorithm, the value of the weight factor gradually decreases. As the curve decreases slowly, the algorithm has a stronger local search ability, which can effectively improve the accuracy of the solution.

In the process of iteration of the algorithm, to avoid the situation that the algorithm cannot jump out of the local optimum in time, the Gaussian mutation operator is introduced to improve the diversity of the population and increase the exploration ability. The Gaussian mutation operator adds the Gaussian perturbation operator based on the initial population, and the Gaussian mutation operator is normally distributed (the mean is $\mu$, and the variance is $\sigma$).

The updating formula of whale individual position after adding Gaussian variation disturbance is:

30 $$ X\left( {t + 1} \right) = X\left( t \right) + X\left( t \right) \cdot \lambda \cdot \delta $$

where $\delta \sim N(0,1)$ is the Gaussian distribution with the mean value of 0 and variance of 1.

31 $$ \lambda = 1 - \frac{t}{{Max_{iter} - 1}} $$

In the formula: when $\lambda = 1$, the variation effect is most significant, while when $\lambda =0$, there is almost no variation.

### Improved genetic whale optimization algorithm flow

**Figure 7:**
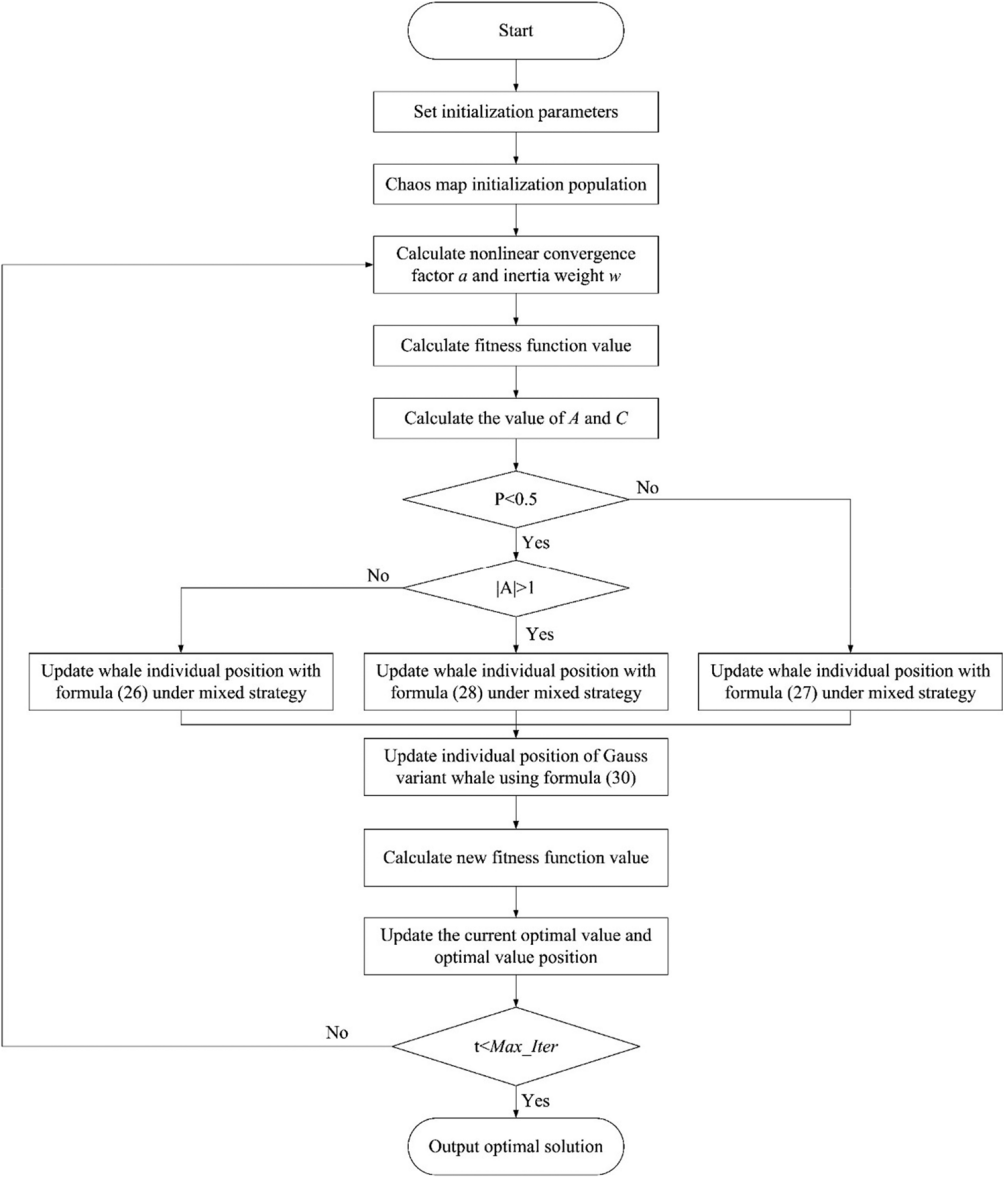
IGWOA flow.

To sum up, the flow of the IGWOA proposed in this paper is shown in Fig. 7.

**Figure:**
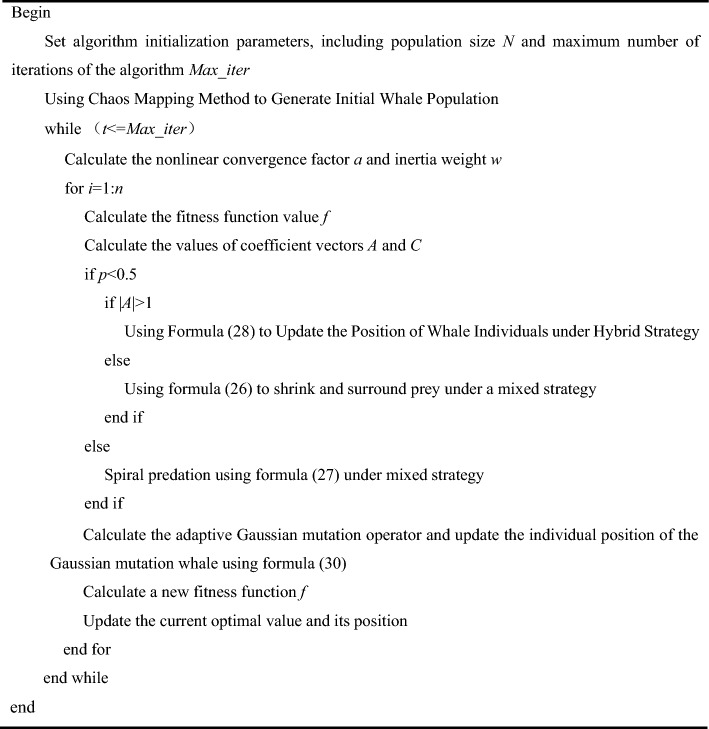
In order to better understand the process of the algorithm, the pseudocode is as follows

## Experimental verification

### Numerical experiment

To fully prove the performance of the IGWOA algorithm, six typical benchmark test functions^38^ of different types are selected for test verification as shown in Table 3. These six benchmark test functions include different types, including (1) single mode functions, (2) complex nonlinear multimodal functions, and (3) fixed low dimensional functions. Through calculation and analysis of different types of test functions, it can show the performance of the algorithm more effectively and comprehensively.

**Table 3:** Benchmark test functions.

Single peak reference function			
Function	Dim	Range	$f_{min}$
$F_{1} = \mathop \sum \limits_{i = 1}^{Dim} \left| {x_{i} } \right| + \mathop \prod \limits_{i = 1}^{Dim} \left| {x_{i} } \right|{ }$	30	[−10, 10]	0
$F_{2} = maxi\left\{ {\left| {x_{i} } \right|,1 \le i \le D} \right\}$	30	[−100, 100]	0

Multimodal reference function			
Function	Dim	Range	$f_{min}$
$F_{3} = \mathop \sum \limits_{i = 0}^{n - 1} x_{i}^{4} + N\left( {0,1} \right){ }$	30	[−1.28, 1.28]	0
$$\begin{aligned} F_{4} & = - 20\exp \left( { - 0.2\sqrt {\frac{1}{Dim}\mathop \sum \limits_{i = 1}^{Dim} x_{i}^{2} } } \right) \\ & \quad - \exp \left( {\frac{1}{Dim}\mathop \sum \limits_{i = 1}^{Dim} {\text{cos}}\left( {2\pi x_{i} } \right)} \right) + 20 + e \\ \end{aligned}$$	30	[−32, 32]	0
$F_{5} = \frac{1}{4000}\mathop \sum \limits_{i = 1}^{Dim} x_{i}^{2} - \mathop \prod \limits_{i = 1}^{Dim} cos\left( {\frac{{x_{i} }}{\sqrt i }} \right) + 1$	30	[−600, 600]	0

Fixed dimensional multimodal reference function			
Function	Dim	Range	$f_{min}$
$F_{6} = \mathop \sum \limits_{i = 1}^{11} \left[ {a_{i} - \frac{{x_{1} \left( {b_{i}^{2} + b_{i} x_{2} } \right)}}{{b_{i}^{2} + b_{i} x_{3} + x_{4} }}} \right]^{2} { }$	30	[−5, 5]	0.00030

**Figure 8:**
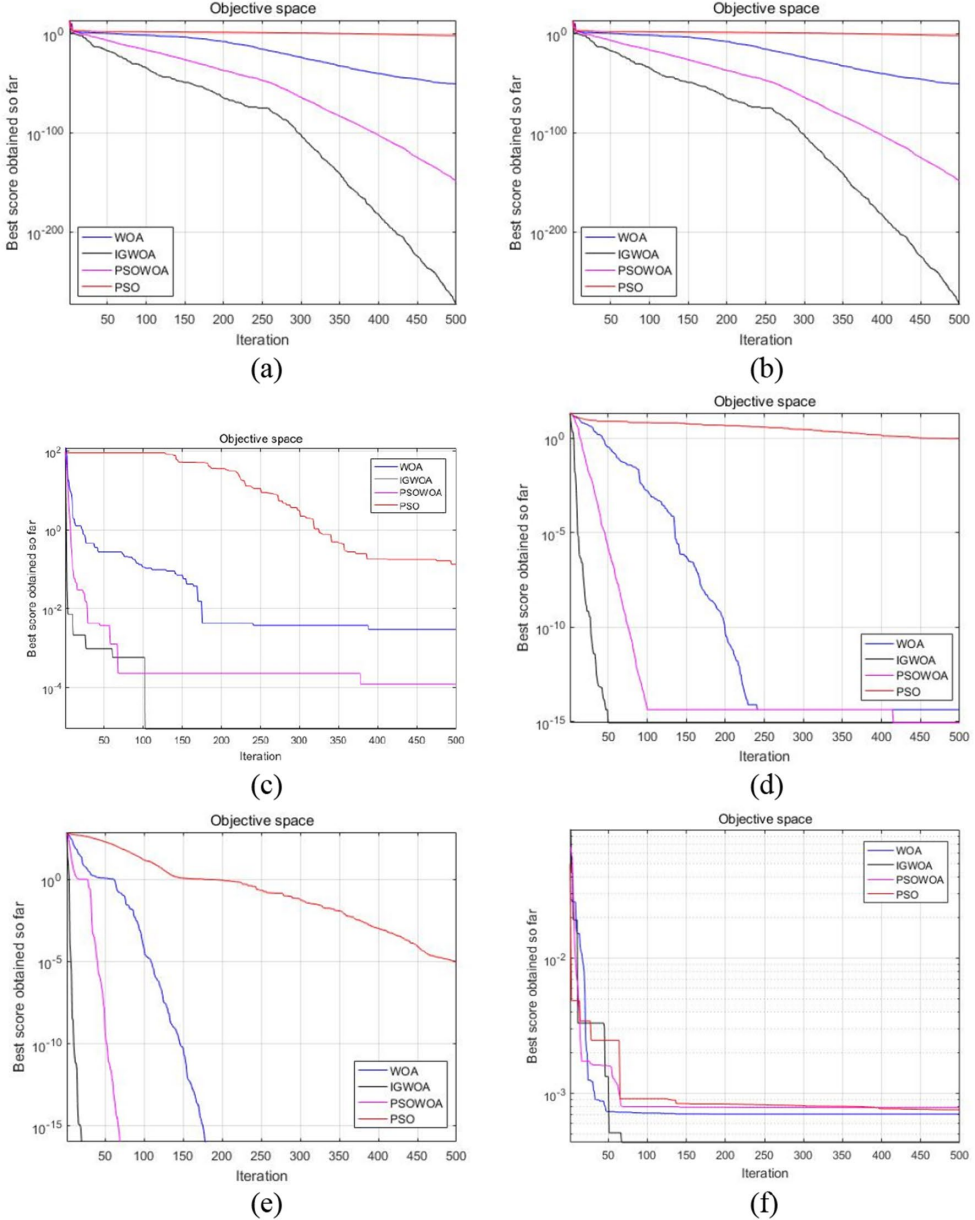
Algorithm convergence diagram.

To verify the performance of the IGWOA algorithm proposed in this paper, several classical and widely used algorithms are selected for comparative analysis. The initial population size of each algorithm is uniformly set to 30, and the maximum number of iterations is set to 500. Each algorithm of WOA, PSOWOA (Particle swarm optimization -Whale optimization algorithm, PSOWOA), PSO (Particle swarm optimization, PSO)^39^, and IGWOA are run separately 30 times. To more clearly show the performance of the IGWOA algorithm proposed in this paper when calculating different types of test functions, the convergence curves of each algorithm when calculating six test functions are plotted as shown in Fig. 8.

From the convergence curve in Fig. 8, it can be seen that in the process of optimizing the six standard test functions, compared with standard WOA, PSO, and PSOWOA, the IGWOA proposed in this paper has the fastest convergence speed, which can effectively save the optimization time of the algorithm. It can also be seen from the figure that compared with the basic WOA algorithm, the ability of the IGWOA algorithm to jump out of the local optimum has been effectively enhanced. Therefore, the IGWOA algorithm has better global search ability than other intelligent algorithms.

For unimodal functions, the search speed of the algorithm is an important indicator to check whether the performance of the algorithm is better. It can be seen from Fig. 8a and b that IGWOA has a faster search speed than other algorithms under the condition of ensuring finding the best global optimization. For multimodal functions, there are many locally optimal solutions, so it is very important to jump out of the local optimal solution and obtain the global optimal solution. As can be seen from Fig. 8c–e, IGWOA can obtain better results on most problems, which can effectively avoid falling into the local optimal solution, to obtain a better global optimal solution. For the fixed dimension multimodal benchmark function, it can be seen from Fig. 8f that the IGWOA algorithm still has better solution efficiency and quality compared with other optimization algorithms. Therefore, when optimizing different types of benchmark functions, the IGWOA algorithm presented in this paper has shown some advantages over other comparison algorithms in terms of both solution accuracy and convergence speed.

### Example verification of shipyard block transportation

**Figure 9:**
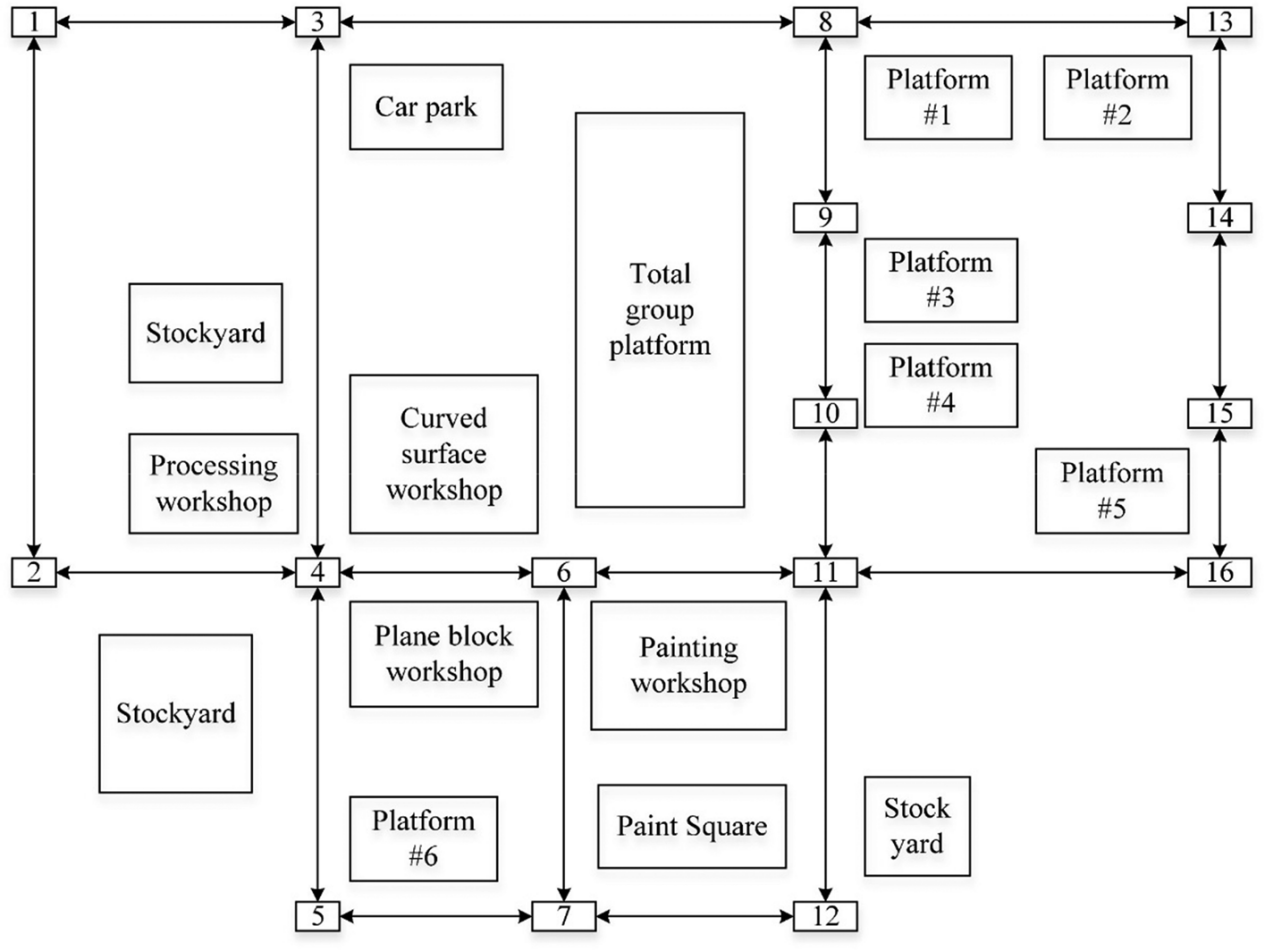
Location distribution of processing workshop and storage yard in a shipyard.

To study the energy-saving ship block Transportation scheduling problem, concerning the actual data of a shipyard, the relevant parameters are set as follows. Figure 9 shows the distribution of each processing plant and yard in a shipyard.

In order to study the energy-saving ship block transportation scheduling problem, referring to the actual data of a shipyard^40^, the relevant parameters are set as follows:

1. At present, the shipyard has 5 types of flatcars, and the information on flatcars of different specifications is shown in Table 4:
2. The weight of blocks is evenly distributed between 100 and 500 t;
3. The block transportation task involves 20 workshops and storage yards of the shipyard, and the distance between workshops or storage yards shall be evenly distributed between 1 and 3 km.
4. The loading and unloading time of blocks at the starting point and end point of the transportation task shall be evenly distributed from 10 to 30 min.

**Table 4:** Parameters of platform trailers.

Load capacity (ton)	100	200	300	400	500
No load speed (m/min)	250	250	200	180	150
Full load speed (m/min)	135	120	100	75	50
Unit fuel consumption without load (l/km)	0.6	1.0	1.5	2.1	2.8
Unit fuel consumption under full load (l/km)	1	2.1	3.0	4.2	5.4

The IGWOA algorithm is applied to solve the example composed of multiple groups of tasks and flatcars. For the objective function, when $\omega_{1} = 1,\; \omega_{2} = 0$, it means that only the flatcar idle time is considered; when $\omega_{1} = 0,\; \omega_{2} = 1$, it means only the flatcar energy consumption is considered; when $\omega_{1} = 0.5,\; \omega_{2} = 0.5$, it means that the flatcar idle time and flatcar energy consumption account for half respectively. The numerical experiment results are shown in Table 5.

**Table 5:** Numerical experimental results.

No.	M*N	$\omega_{1} = 1,{ }\omega_{2} = 0$		$\omega_{1} = 0,{ }\omega_{2} = 1$		$\omega_{1} = 0.5,{ }\omega_{2} = 0.5$	
		*T*_1_ (min)	*E*_1_ (l)	*T*_2_ (min)	*E*_2_ (l)	*T*_3_ (min)	*E*_3_ (l)
1	10*2	155.2	42.4	175.2	33.5	170.2	37.5
2	10*3	195.3	53.4	215.3	41.3	210.3	46.3
3	20*3	282.7	76.5	302.7	59.7	297.7	67.1
4	20*4	305.2	84.9	325.2	64.6	318.6	75.9
5	30*5	424.8	156.3	454.8	121.3	439.8	142.6
6	30*6	447.5	182.6	484.1	143.6	469.6	162.5
7	40*6	536.8	293.2	586.8	219.8	561.3	256.1
8	40*7	560.4	342	603.5	263.2	589.3	302.6
9	50*7	657.7	509.9	700.6	388.4	684.1	448.9
10	50*8	680.4	563.4	726.8	426.1	708.6	491.4

**Figure 10:**
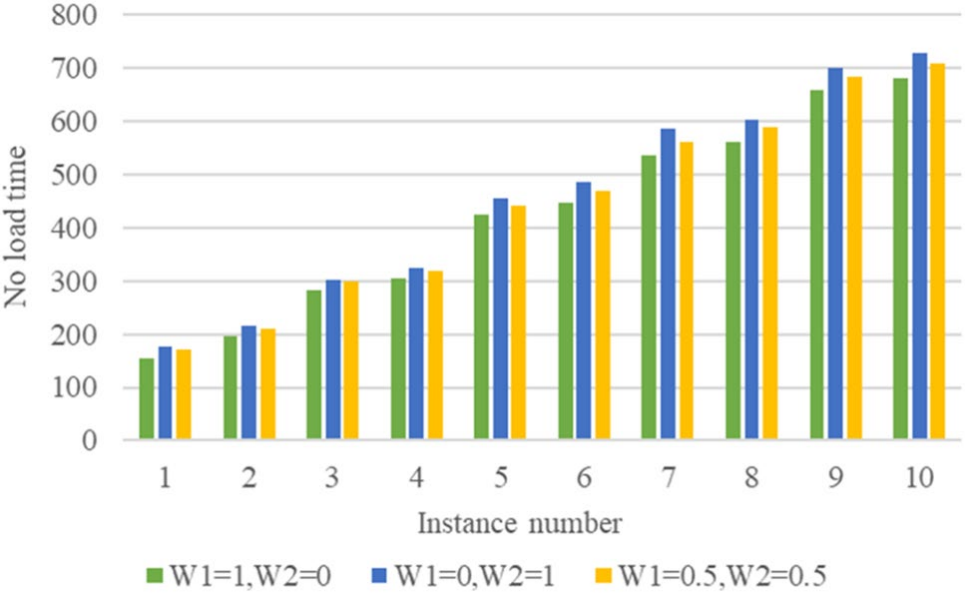
No load time of flat cars in 10 cases under different weights.

**Figure 11:**
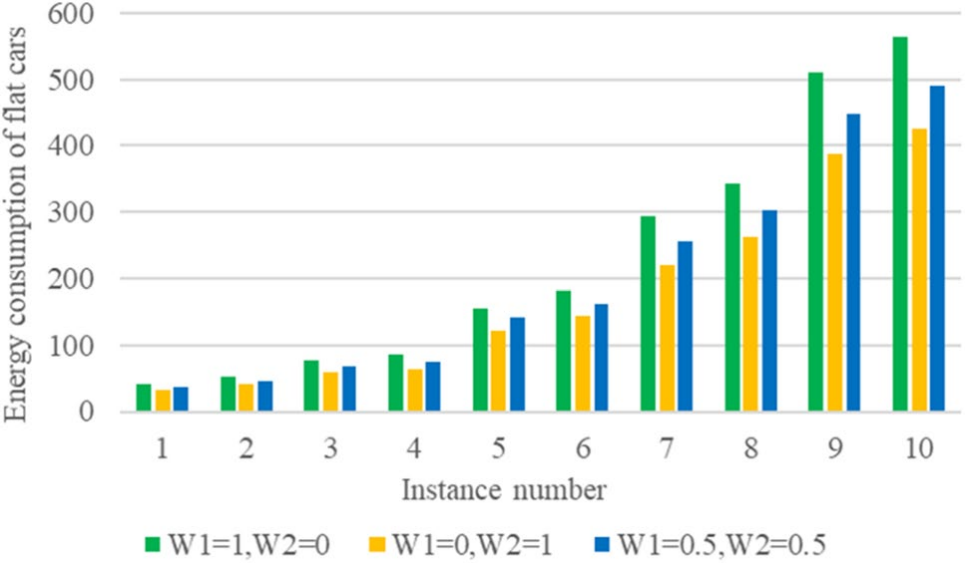
Energy consumption of flat cars in 10 cases under different weights.

Collating the above data and drawing bar charts, as shown in Figs. 10 and 11, can show more visually the differences in the values of each objective function for different size instances with different weights.

$T_{1}$ represents the flatcar no-load time value considering only the flatcar no-load time scheduling scheme, $E_{1}$ represents the flatcar energy consumption value considering only the flat car no-load time scheduling scheme, $E_{2}$ represents the flat car energy consumption value considering only the flatcar energy consumption scheduling scheme, $T_{2}$ represents the flatcar no-load time value considering only the flatcar energy consumption scheduling scheme, $E_{3}$ represents the flatcar energy consumption value of the scheduling scheme when $\omega_{1} = 0.5,{ }\omega_{2} = 0.5$, $T_{3}$ represents the no-load time value of the flatcar in the dispatching scheme when $\omega_{1} = 0.5,{ }\omega_{2} = 0.5$. According to the calculation results, it can be seen that the value of flatcar no-load time and flat car energy consumption increases with the increase of the problem scale. In addition, as shown in Table 6, compared with the scheduling scheme that only considers the empty time of flatcars, choosing the scheduling scheme that only considers the energy consumption of flatcars will increase the empty time of flatcars by less (the calculation formula is $\left( {T_{2} - T_{1} } \right)/T_{1}$), but will reduce the energy consumption of flatcars by more (the calculation formula is $\left( {E_{1} - E_{2} } \right)/E_{1}$). That is to say, the proportion of the reduction of energy consumption of flatcars is far greater than the proportion of the increase of empty time.

**Table 6:** Comparison of index improvement.

No.	M*N	$\omega_{1} = 0,{ }\omega_{2} = 1$		$\omega_{1} = 0.5,{ }\omega_{2} = 0.5$	
		$\left( {T_{2} - T_{1} } \right)/T_{1}$ (%)	$\left( {E_{1} - E_{2} } \right)/E_{1}$ (%)	$\left( {T_{3} - T_{1} } \right)/T_{1}$ (%)	$\left( {E_{1} - E_{3} } \right)/E_{1}$ (%)
1	10*2	12.89	21.43	9.66	11.90
2	10*3	10.24	22.64	7.68	13.21
3	20*3	7.07	22.37	5.31	11.84
4	20*4	6.55	23.81	4.39	10.71
5	30*5	7.06	22.44	3.53	8.97
6	30*6	8.18	21.43	4.94	10.71
7	40*6	9.31	25.26	4.56	12.63
8	40*7	7.69	23.10	5.16	11.55
9	50*7	6.52	23.77	4.01	11.89
10	50*8	6.82	24.33	4.14	12.70

**Figure 12:**
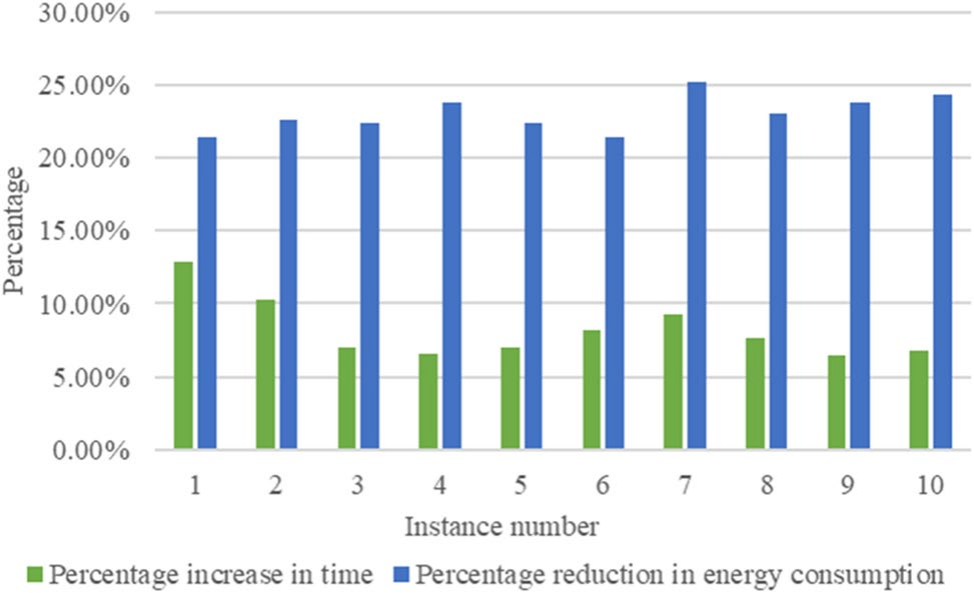
Index improvement comparison of 10 cases when $\omega_{1} = 0,\;\omega_{2} = 1$.

**Figure 13:**
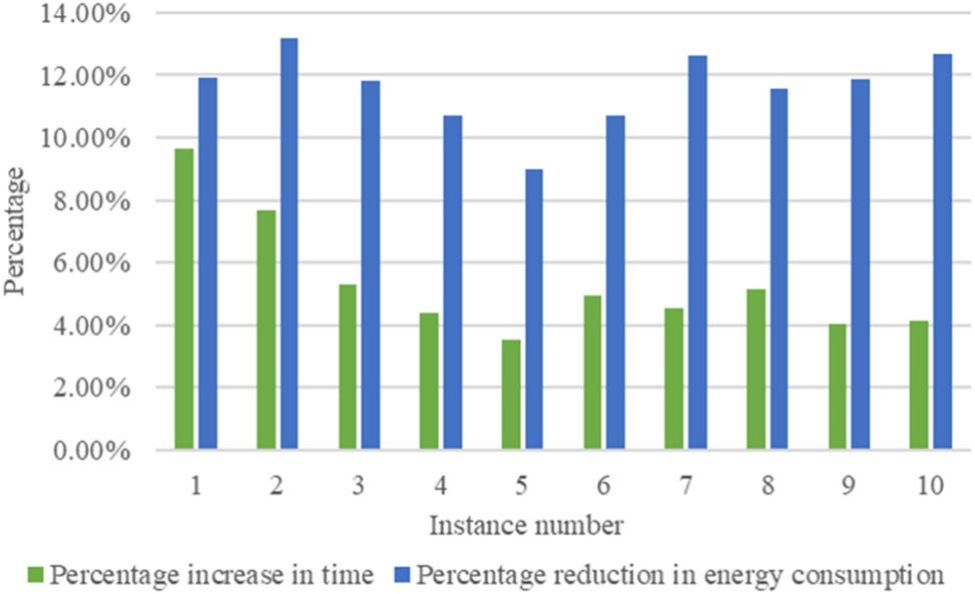
Index improvement comparison of 10 cases when $\omega_{1} = 0.5,\;\omega_{2} = 0.5$.

Collating the above data and drawing bar graphs, as shown in Figs. 12 and 13, can show more intuitively the comparison of each instance in terms of index improvement under different weights, which can show the practicality of the energy-saving-oriented ship block transportation scheduling method proposed in this paper.

## Conclusions

In order to reduce the energy consumption of the ship block transportation process, the empty time and energy consumption of the flatcars during the block transportation process are taken as the optimization objectives and the corresponding scheduling model is established, and an improved genetic whale optimization algorithm is proposed. According to the validation results of the shipyard segment transportation example, it is shown that under the constraint of meeting the hard time window of the transportation task, compared with the scheduling scheme considering only the flatbed truck unloading time, choosing the scheduling scheme considering only the flatbed truck energy consumption will improve less flatbed truck unloading time but reduce more flatbed truck energy consumption, which means the ratio of energy consumption reduction is greater than the ratio of time increase. Thus, it proves the engineering practicality of the green dispatching method proposed in this paper, which can further provide a decision-making method for shipyard managers.

To sum up, the following conclusions can be drawn:

1. To reduce the energy consumption in the process of ship block transportation, the no-load time and energy consumption of flat cars in the process of block transportation are taken as the optimization objectives, the corresponding scheduling model is established, and an IGWOA is proposed.
2. The use of an optimization algorithm to schedule flatcars is fast, which can greatly improve the current situation of resource waste and disorder caused by the real-time manual distribution of flatcars, and make full use of flatcar resources in the yard.
3. According to the verification results of the shipyard’s block transportation example, under the constraint of meeting the hard time window of the transportation task, compared with the scheduling scheme that only considers the flatcar’s empty time, choosing the scheduling scheme that only considers the flatcar’s energy consumption will increase the flatcar’s empty timeless, but will reduce the flatcar’s energy consumption more, that is, the proportion of energy consumption reduction will be greater than the proportion of time increase, It proves the engineering practicability of the energy-saving ship block transportation scheduling method proposed in this paper.

In future research, more specific issues related to green vehicle scheduling need to be studied, such as multi vehicle collaborative scheduling, green scheduling of heterogeneous vehicles, dynamic scheduling of green vehicles in uncertain environments, and so on. More and more advanced optimization algorithms are being proposed (such as alternative hybrid heuristics and meta heuristics, adaptive algorithms, adaptive algorithms, islanding algorithms, polyploid algorithms, hyper heuristics)^41–45^, and have been used in different fields as solutions. Therefore, in the future, it is necessary to further explore the effectiveness of advanced optimization algorithms in the application of this problem, and compare the method proposed in this paper with more advanced optimization algorithms.

## Data Availability

The authors confirm that the data supporting the findings of this study are available within the article.
